# RNA-Seq optimization with eQTL gold standards

**DOI:** 10.1186/1471-2164-14-892

**Published:** 2013-12-17

**Authors:** Shannon E Ellis, Simone Gupta, Foram N Ashar, Joel S Bader, Andrew B West, Dan E Arking

**Affiliations:** 1 McKusick-Nathans Institute of Genetic Medicine, Johns Hopkins University School of Medicine, Baltimore, USA; 2 Department of Biomedical Engineering, Johns Hopkins University School of Medicine, Baltimore, USA; 3 Department of Neurology, University of Alabama School of Medicine, Birmingham, USA

**Keywords:** eQTL, Brain, Blood, LCL, RNA-Seq, GTEx

## Abstract

**Background:**

RNA-Sequencing (RNA-Seq) experiments have been optimized for library preparation, mapping, and gene expression estimation. These methods, however, have revealed weaknesses in the next stages of analysis of differential expression, with results sensitive to systematic sample stratification or, in more extreme cases, to outliers. Further, a method to assess normalization and adjustment measures imposed on the data is lacking.

**Results:**

To address these issues, we utilize previously published eQTLs as a novel gold standard at the center of a framework that integrates DNA genotypes and RNA-Seq data to optimize analysis and aid in the understanding of genetic variation and gene expression. After detecting sample contamination and sequencing outliers in RNA-Seq data, a set of previously published brain eQTLs was used to determine if sample outlier removal was appropriate. Improved replication of known eQTLs supported removal of these samples in downstream analyses. eQTL replication was further employed to assess normalization methods, covariate inclusion, and gene annotation. This method was validated in an independent RNA-Seq blood data set from the GTEx project and a tissue-appropriate set of eQTLs. eQTL replication in both data sets highlights the necessity of accounting for unknown covariates in RNA-Seq data analysis.

**Conclusion:**

As each RNA-Seq experiment is unique with its own experiment-specific limitations, we offer an easily-implementable method that uses the replication of known eQTLs to guide each step in one’s data analysis pipeline. In the two data sets presented herein, we highlight not only the necessity of careful outlier detection but also the need to account for unknown covariates in RNA-Seq experiments.

## Background

The advent of RNA-Seq [1] and dramatic decrease in next-generation sequencing costs have led to numerous RNA-Seq studies in recent years. This revolutionary technique has enabled digital transcriptome profiling at unprecedented resolution that avoids many of the limitations inherent to the analog nature of microarray technology [2,3]. However, despite numerous publications and the fact that RNA-Seq studies have supplanted microarrays as the gold standard for transcriptome analysis, it is not without its own inherent limitations.

**Figure 1:**
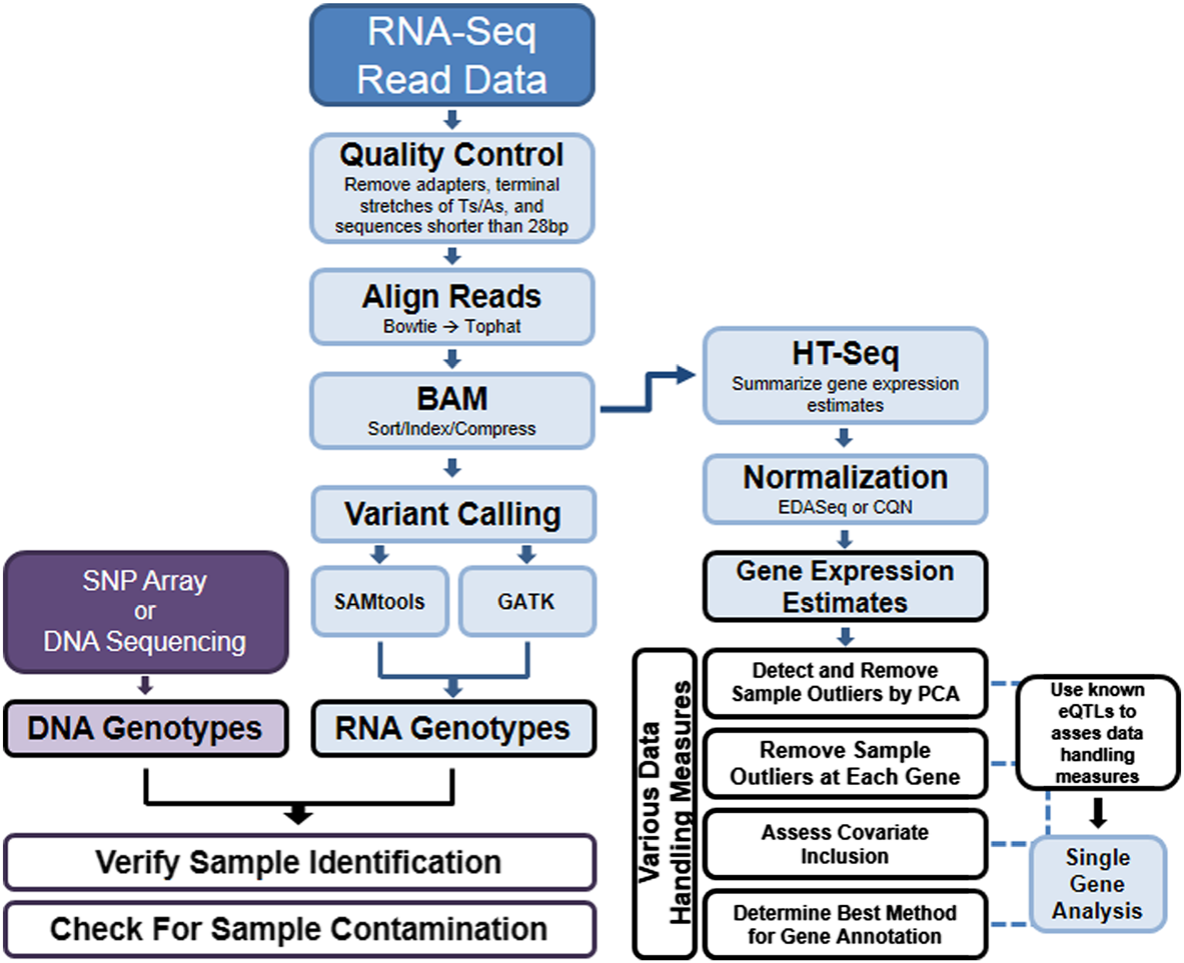
**Data analysis pipeline for analysis of RNA-Seq data.** Blue boxes are data analyses carried out on RNA. Purple indicates DNA.

Early concerns regarding library preparation, sequencing error, read mapping, and gene expression quantification have been resolved by a number of studies; however, there is no standardized approach for quality control and data adjustment of RNA-Seq data after the generation of gene expression estimates. Without an appropriate approach to data analysis, reproducibility of these studies remains limited [4]. Further, the unique designs of sequencing studies suggest that a single black box approach is unlikely to be uniformly optimal across all experiments. Thus, we propose an approach to address data cleaning, normalization, and adjustment in RNA-Seq data analysis (Figure 1). This pipeline is informed by best practices that we and others have developed for genome-wide association studies (GWAS) [5,6], which also suffered from similar sources of error prior to the development of optimized methods.

We demonstrate the applicability of our approach in 64 autism-affected and control brain samples. Specifically, our outlier detection method is based on utilizing the RNA-Seq gene expression estimates as well as DNA and RNA genotypes obtained from the same individual. Further, expression quantitative trait loci (eQTLs) are biologically meaningful loci at which gene expression is modified by genotype. Accordingly, we utilize replication of *cis*-eQTL data from two recently published brain studies [7,8] as a means to assess the integrity of sequencing data and appropriateness of data handling procedures. We replicate the findings from this eQTL analysis in an independently-generated RNA-Seq data set of 162 blood samples from the Genotype-Tissue Expression (GTEx) project [9]. Within the context of eQTL replication, we particularly highlight the need to identify and remove outlier samples in RNA-Seq experiments and further corroborate the necessity of accounting for unknown sources of variation in high-throughput data [10]. While a number of publications have presented methods by which one can analyze RNA-Seq data (many of which are reviewed in [11]) and account for unknown covariates [12-15], the steps we present herein ultimately provide a straightforward approach that allows for more accurate approximation of gene expression values that can be confidently used in downstream disease-based comparisons.

## Results

### Data normalization in RNA-Seq

**Figure 2:**
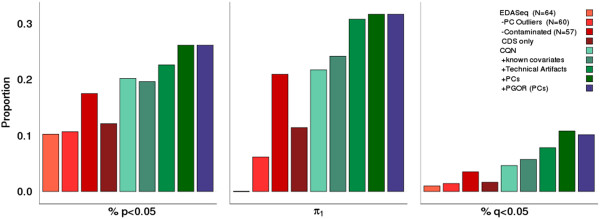
**eQTL replication in brain data.** Normalization by EDASeq (red bars) demonstrates that sample outlier removal improves eQTL replication and that whole gene annotation provides improved gene expression estimation than coding sequence (CDS) only. CQN normalization (green bars) provides slightly improved eQTL replication over EDASeq normalization and demonstrates the necessity of covariate inclusion in eQTL replication, particularly highlighting the necessity of accounting for unknown covariates. Per-gene outlier removal (PGOR, blue bars) does not hamper our ability to detect *cis*-eQTLs.

Brain RNA-Seq data were generated from post-mortem cortical samples collected from Brodmann Area 19 (BA19) in 39 control and 25 autism-affected cases (see Additional file 1: Table S1). After estimating gene expression from the sequencing reads, two methods for data normalization were assessed: Exploratory Data Analysis and Normalization for RNA-Seq (EDASeq) [16] and Conditional Quantile Normalization (CQN) [17]. The normalized gene expression values from each algorithm demonstrated method-specific biases. Examining p-values from our covariate adjusted case–control analysis, we note that normalization by CQN leads to a marked increase in the test statistics for shorter and low GC content genes (gene length < 1000 bp, GC content < 35%), a problem not observed with EDASeq (see Additional file 1: Figure S1). On the other hand, genes with both lower gene expression estimates and the assignment of zero values by EDASeq led to an increase in outliers on a per-gene basis in our eQTL analyses (see Additional file 1: Figure S2A), whereas CQN did a better job handling these genes (see Additional file 1: Figure S2B). Further comparison by eQTL replication to assess the biologic reproducibility (discussed below) of these two normalization methods was performed with CQN slightly outperforming EDASeq (Figure 2). While one unified approach that directly addresses the limitations of each approach more effectively would improve results, we selected CQN for downstream analyses due to its slight improvement in eQTL replication. Nonetheless, we recommend that, until the presented issues are directly addressed, both methods be considered as part of an analysis pipeline.

### Identifying outliers in RNA-Seq data

In large sequencing studies, specific samples, for technical or biological reasons, can be recognized as outliers and should be removed from the study [18]. To identify outlier samples, whose global gene expression pattern is not explained by known covariates, we used Principal Component Analysis (PCA), investigating the first six principal components, which together explain ~60% of the variance in the brain data. Samples greater than three standard deviations (SD) from the mean in any of the first six principal components were deemed outliers and removed from analysis (N = 4 or 6.3% of all samples) (see Additional file 1: Figure S3).

After sample-based outlier removal described above, it was apparent that, on a gene-by-gene basis, there were samples whose expression estimates differed greatly from the rest of the samples for that particular gene (see Additional file 1: Figure S2). Using a cut-off of three SD from the mean, 20.2% [7,027/34,738] of genes tested for differential expression between cases and controls had at least one sample flagged as an outlier for gene expression level. As these sample outliers are gene-specific, they suggest a clear artifactual origin, as opposed to a problem with the sample as a whole. Comparing the 50 most significantly differentially expressed genes between cases and controls before and after outlier removal, the lists differ at 60% [30/50] of the genes present (see Additional file 2), demonstrating that inaccurate results would be reported if gene-by gene outliers were not removed. To further ensure that this was indeed biologically sound, we assessed the validity of this approach using our eQTL analysis (discussed below).

After flagging outlier samples for removal in the brain data set, we obtained genotypes from both DNA and RNA. As a check on our data, we verified sample identity by comparing each RNA-Seq sample against all DNA samples. Pair-wise Identity by State (IBS) distances (DSTs) were calculated in PLINK with the expectation that DNA and RNA genotypes generated from the same individual should have a DST value approaching 1.0. In all samples, DNA genotypes best matched their corresponding RNA genotypes with a DST > 0.83, indicating that our DNA and RNA samples were, in fact, from the same individual.

Despite correct identification of sample identity by IBS, three samples had borderline DST values (DSTs = 0.83-0.89), warranting further investigation. These samples demonstrated an unexpected genotyping comparison profile such that all three showed an increased number of genotype calls deemed homozygous by DNA genotyping but called heterozygous at the RNA level. As DNA genotyping by Affymetrix array has proven to be extremely accurate [19], an excess of sites where the DNA genotype indicates homozygosity but heterozygous calls are present at the RNA level indicates possible contamination. We quantify these occurrences in each sample using a metric we refer to as the Discordance Ratio (DR). For the majority of our samples, for which there is no suspected contamination, the DR approaches zero, with a value less than 0.2 indicating RNA-Seq data of sufficient quality for further analysis. The three samples in question had elevated DRs (0.32, 0.41, and 0.47), suggestive of sample cross-contamination (see Additional file 1: Figure S4).

**Figure 3:**
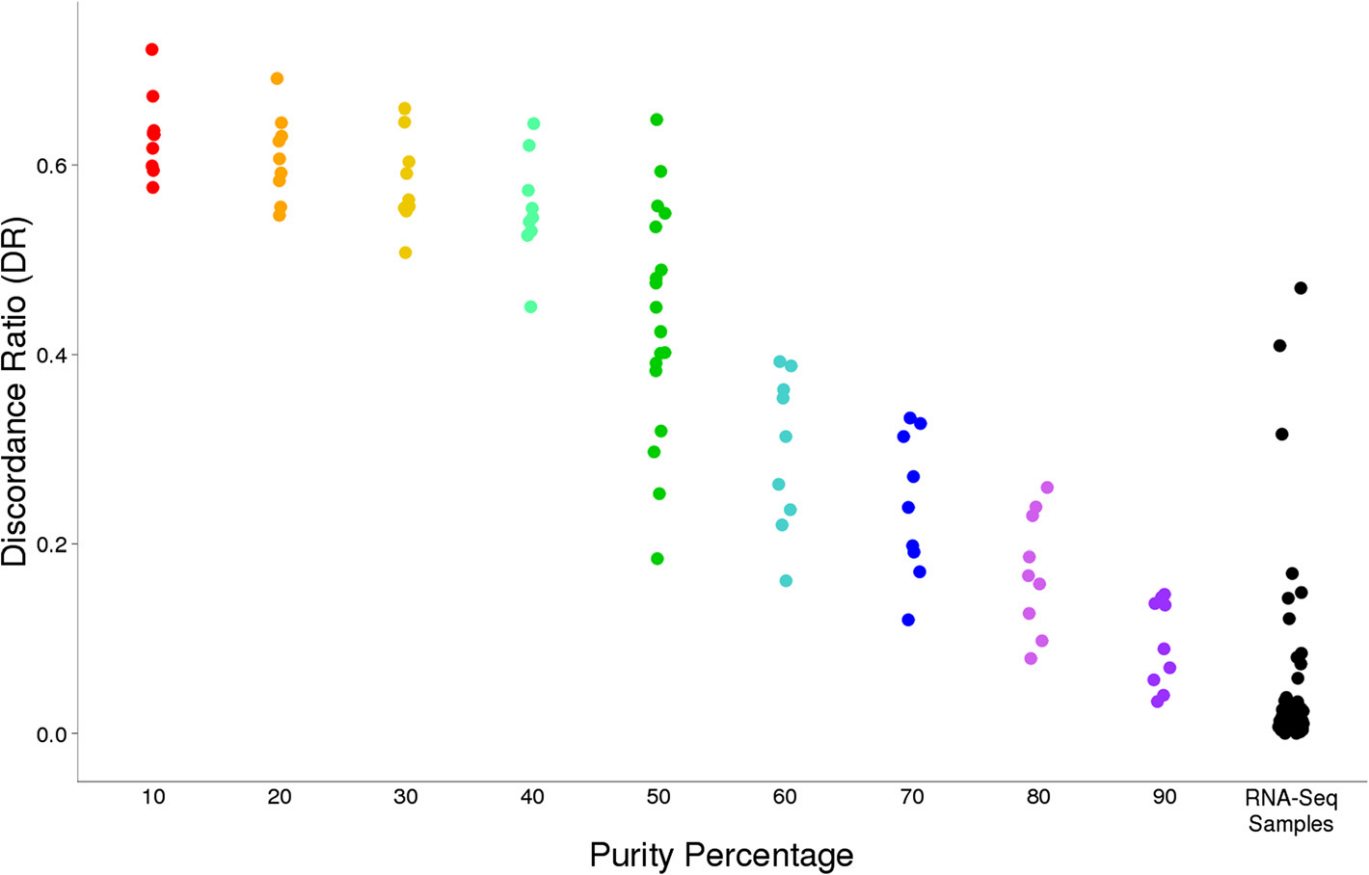
**Simulated contamination of RNA-Seq reads.** Sample contamination was simulated by mixing RNA-Seq reads from two different samples in controlled ratios. These intentionally contaminated samples’ RNA genotypes were then compared back to the DNA genotypes from which they were mixed. Specifically, a Purity Percentage of ‘10’ indicates that 10% of the reads in that RNA genotype file were sampled from the DNA sample to which it was compared.

To address the possibility of contamination, we conducted a mixing experiment where we combined high quality RNA-Seq samples (identified as having a DR < 0.1) in controlled ratios. We carried out variant calling on these intentionally contaminated samples as had been previously carried out in the RNA-Seq data and calculated the DR for each. This ratio was then compared between the RNA-Seq samples in question and those from which mixing had been simulated. This comparison suggests that, for the three sample libraries in question, 30-70% of the RNA-Seq reads originated from a different sample (Figure 3). As reads from a foreign sample would lead to inaccurate gene expression estimates, we removed these samples from downstream analysis, resulting in a final data set of 57 samples, comprising 21 controls and 36 cases.

### Reported brain eQTLs are reproducible in RNA-Seq data

Previously, surrogate measures of RNA quality (e.g., pH, post-mortem intervals, RIN values, etc.) have been used in an attempt to predict biologic validity, but none has been uniformly successful. Using published sets of brain eQTLs – regulatory genomic loci at which gene expression levels in the brain differ by genotype – we looked to recapitulate a number of the previously reported brain eQTLs in our gene expression data. We postulated that if we could replicate these eQTLs in our data, this would indicate that the use of post-mortem brain tissue may be representative of physiological conditions. We used a list of 909 *cis*-eQTLs generated from two recent studies that detected brain eQTLs in multiple disease populations across a number of brain regions [7,8] (see Additional file 3). Despite a smaller sample size and only one brain region under interrogation, we replicate 26.1% [237/909] of the tested associations (inflation-adjusted p < 0.05) when age, sex, site and principal components are included as covariates (Figure 2 & see Additional file 1: Figure S5 & Table S2).

### Monitoring eQTL replication to gauge quality control measures

We posit that if we are appropriately handling our data, known brain eQTLs should demonstrate improved association after each data correction step as well as an overall increase in the number of previously reported eQTLs that replicate. We have measured the ability to replicate known *cis*-eQTLs associations using three metrics: (1) the percentage of known eQTLs that replicate at p < 0.05 after adjusting for genome-wide inflation (See Methods) (2) π_1_, a statistic that estimates of the proportion of significant tests [20], and (3) the percentage of known eQTLs that replicate at q < 0.05. When taken together, these three metrics offer a profile of the validity of each data handling step.

As part of the initial quality control, seven of the 64 samples (11% of total) were flagged as PCA outliers or contaminated samples, and removed. To assess the effect of sample removal, we compared eQTL replication in three data sets: (1) prior to outlier removal (N = 64), (2) after dropping PC outliers (N = 60), and (3) after dropping likely contaminated samples (N = 57). Sample outlier removal allows for the detection of 7.4% more known eQTLs p < 0.05 and 3.5% more eQTLs q < 0.05. Similarly, π_1_ estimates a dramatic increase in the proportion of replicating eQTLs from 0.000 to 0.209. These data indicate the necessity of removing suspect samples in these data (Figure 2 & see Additional file 1: Table S2).

We further utilized eQTL replication to determine the most appropriate model for gene annotation. There is evidence that suggests expression levels estimated from RNA-Seq data at the coding sequence (CDS) alone correspond better with qRT-PCR measurements than RNA-Seq estimates that include both the CDS and its untranslated regions (UTRs). However, recent RNA-Seq analyses have generally included gene annotation from the whole gene – that is the CDS and its UTRs – under the argument that gene annotation gains accuracy upon UTR inclusion [21]. To address this discrepancy in the literature, we compared these two gene annotation approaches by eQTL replication. The whole gene annotation clearly replicates known eQTLs better than the CDS alone (Figure 2 & see Additional file 1: Table S2) detecting 5% more known eQTLs at p < 0.05 and 1.9% more at q < 0.05. Replication, as measured by π_1_ demonstrates an increase in this test statistic as well (0.114 in CDS, 0.209 in whole gene annotation). This improvement in eQTL detection offers support for the use of UTR inclusion in gene annotation in these data.

Similarly, eQTL replication was used to compare normalization methods. We note that when considering the overall number of known eQTLs detected, CQN replicates 2.7% more eQTLs (p < 0.05) than does EDASeq (Figure 2 & see Additional file 1: Table S2), further supporting its use in analyzing gene expression in this data set.

Disease-based comparisons are frequently adjusted for known covariates (age, sex, etc.). However, comparative studies are also frequently plagued by unknown covariates, or confounders within the data that are not easily attributable to any recorded measurement [10,18]. These unknown covariates can be approximated through various data decomposition methods. We initially considered using PCA to accomplish this goal but observed that the first PC was correlated with both collection site (see Methods) and disease status, which often occurs whenever different sites have differing fractions of cases and controls. As this could be a likely issue in many case–control studies, limiting the utility of PCs in downstream analyses, we also considered Surrogate Variable Analysis (SVA) [14] and Independent Surrogate Variable Analysis (ISVA) [15], as these approaches allow for disease status to be protected during their generation. Lastly, we also considered utilizing PEER [13,22] to account for unknown covariates, as this method has been used and performed well in previous eQTL analyses [23]. In eQTL replication analyses, performance was comparable with ISVs, SVs, PEER and PCs detecting 25.1, 26.2, 26.9 and 26.1 percent of the previously reported eQTLs, respectively (p < 0.05) (see Additional file 1: Table S2). Ultimately, however, to address the case–control confounding issue, we had to decide between ISV and SV usage. To do so, we tested both methods by assessing Q-Q Plots generated for disease-based comparisons. As the inclusion of SVs, but not ISVs, demonstrated inflated p-values in these analyses (see Additional file 1: Figure S6), we decided to move forward with ISVs to account for unknown covariates.

Finally, regarding covariate inclusion, we note that certain metrics for technical artifacts of sequencing (percent coding bases, percent intronic bases, percent mRNA bases, median 3′ bias, percent UTR bases, and AT dropout) were correlated with specific ISVs (see Additional file 1: Table S4), suggesting that the unknown covariates detected by ISVA may simply be accounting for known technical artifacts of sequencing. We tested this possibility and demonstrate that, while including technical artifacts as covariates does improve eQTL detection over known covariates alone (2.4% increase at p < 0.05, increase in π_1_ from 0.217 to 0.308), both PCs and ISVs perform even better, demonstrating a 5.9% and 4.9% increase at p < 0.05, respectively, when compared to no covariate inclusion (Figure 2 & Additional file 1: Table S2). These data ultimately support the inclusion of covariates, as captured by data decomposition methods, in downstream analyses suggesting that such methods are either (a) accounting for unknown covariates beyond technical sequencing artifacts or (b) appropriately weighting the effects of the technical artifacts amongst the ISVs/PCs generated.

As noted above, sample outliers were also identified on a per-gene basis and removed from analysis. To ensure that removing these outliers was biologically sound and that these outliers did not represent true measures of differential expression, we tested data sets where sample outliers were removed at each gene using our eQTL replication approach. While per gene outlier removal did not demonstrate a marked increase or decrease in eQTLs detected (Figure 2 & Additional file 1: Table S2), the presence of outlier samples leads to a lack of robustness in the case–control analysis where single samples dramatically skewed the results (see Additional file 1, Figure 2). As per-gene outlier removal helped to stabilize the case–control analyses and did not hinder our ability to detect known eQTLs, we support its inclusion in RNA-Seq data analysis.

### Independent RNA-Seq data set supports use of eQTL gold standards

**Figure 4:**
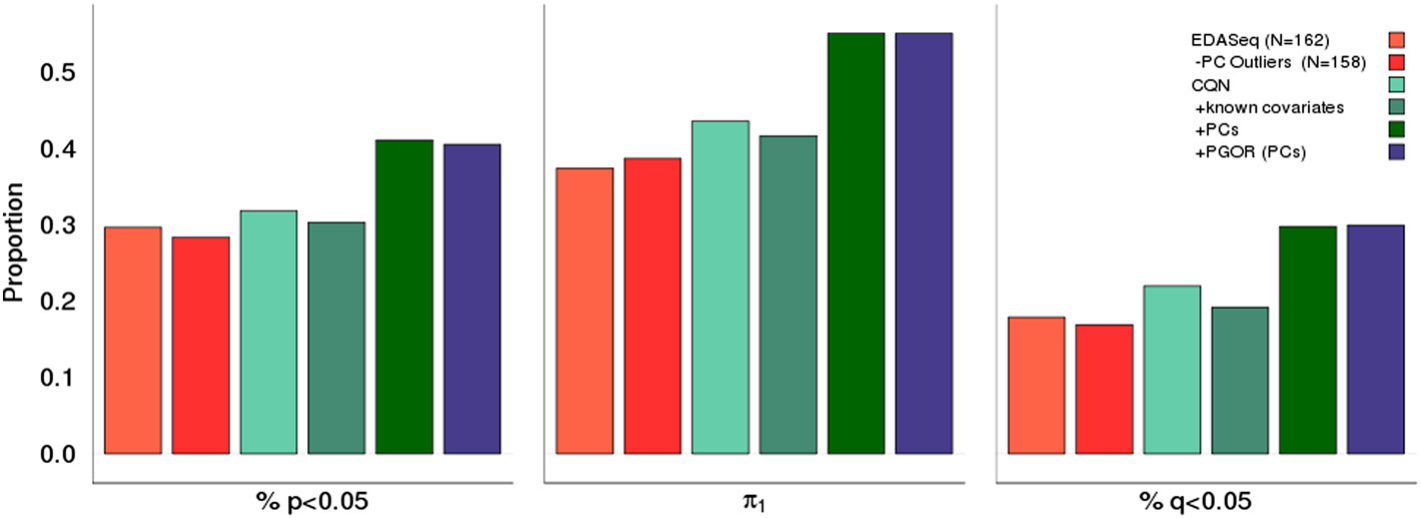
**eQTL replication in blood data.** Colors correspond to the comparable analyses carried out in the brain data (Figure 2). Again, these data show that CQN (green bars) slightly improves eQTL detection over EDASeq (red bars) and that a considerable increase in eQTL detection is seen when unknown covariates are considered in the analysis.

To bolster the results of our brain RNA-Seq data set, we set out to replicate the main findings of our initial analysis in an independent RNA-Seq data set generated from a distinct tissue source. To do this, we used 162 blood samples from the GTEx project [9], for whom we had DNA genotypes as well as raw count data from RNA-Seq. In these data, four samples (2.5% of total) were identified as PC outliers, using the same criteria as was used in the brain data. Sample outlier removal led to a slight decrease in the number of eQTLs detected (29.6% versus 28.3% at p < 0.05); however, there was an increase in π_1_ (0.374 to 0.387 after outlier removal) (Figure 4 & Additional file 1: Table S3). Normalizing using CQN again led to an overall increase in eQTLs detected (3.5% increase at p < 0.05) (Figure 4 & Additional file 1: Table S3). In assessing covariate addition, a pattern similar to what was seen in the brain data was observed. While known covariates (age, sex, and cohort) in the brain data did not improve the eQTL detection, there was a similar improvement seen upon the addition of PCs to account for unknown covariates (9.3% increase when compared to the use of no covariates) (Figure 4 & Additional file 1: Table S3). Again, per gene outlier removal does not hamper the ability to detect known eQTLs (Figure 4 & Additional file 1: Table S3).

## Discussion

Just as it took more than ten years for the field to reach a consensus on the analysis of microarray data, RNA-Seq analysis has been undergoing a similar struggle since the first RNA-Seq publication. Since then, accurate library preparation, appropriate mapping of short sequencing reads, and correct estimation of gene expression values have been the primary focus. While many of the original experimental and data analysis hurdles have been addressed, a framework in which one can assess the quality control and data adjustment measures taken after obtaining accurate gene expression estimates was lacking. For experiments with RNA-Seq data, DNA genotypes and a list of tissue-appropriate eQTLs, we demonstrate that our approach can be easily employed to generate a clean set of expression estimates for downstream analyses (Figure 1). Specifically, we propose using the ability to replicate eQTLs as a biologically meaningful check on the integrity of the data and to help ensure that the data is being handled appropriately at each quality control and data adjustment step.

We demonstrate that upon generating normalized gene expression estimates, PCA can be utilized to identify global gene expression sample outliers and that DNA and RNA genotypes can be employed to verify sample identity and check for sample contamination. Using the replication of known eQTLs, we also demonstrate the importance of including both known and unknown covariates in downstream analyses. This result is consistent with expectation [18], illustrating the utility of eQTL replication as a simple approach to assess data handling measures and offering credence to its usage in additional comparisons. This eQTL replication approach was further employed to demonstrate that ISVs are not simply explaining known technical artifacts of next-generation sequencing, that gene annotation best replicates known biology when UTRs are included in gene annotation, and that we do not overtly lose power to detect known eQTLs when reducing the sample size by removing suspect samples.

Of the data cleaning steps employed, we highlight the importance of removing individuals on a per-gene basis, as this is not a standard quality control step. Indeed, either due to low coverage (leading to zero counts) or undetected PCR duplication (leading to an overabundance of counts), a sample may exhibit gene expression values vastly different than the rest of the samples, and as a result, should be removed from analysis at that particular gene prior to ISV/PC generation. We note that this process is more important when using EDASeq than when CQN is used for normalization, as quantile normalization produces fewer outlier values. In the brain data set, when EDASeq was employed, 42.5% of genes had at least one sample flagged as an outlier (see Additional file 1: Figure S2A), whereas the nature of quantile normalization produced outliers in only 20.2% of genes tested. Nevertheless, with both methods, the differential gene expression analysis becomes more robust upon the removal of individual samples present in the data skewing the results (see Additional file 1: Figure S2B). While these sample-specific outlier genes could certainly reflect an insertion-deletion event or another genetic variation in these individual samples, our goal is to maximize one’s ability to find eQTLs and assess overall data handling measures, and as such, these individuals should be removed from analysis. Given that our ability to detect eQTLs is not hampered and that differential gene expression analysis demonstrates improved robustness upon per-gene outlier removal, we argue that this novel outlier identification approach be incorporated in future RNA-Seq expression studies.

The utilization of two distinct RNA-Seq data sets – one generated from brain (N = 64) and another independently generated from blood (N = 162) – helps to demonstrate the main findings of this work. In both brain and blood data sets, eQTL replication was improved with the use of CQN for data normalization and further improved upon the addition of PCs as covariates. Further, in both data sets, removing per-gene outliers did not hamper the ability to detect known eQTLs. However, there are data handling measures imposed on the data that were more important in the brain data set than in the blood data set, likely reflecting the fact that the brain data was plagued by a smaller sample size and that the sequencing data was generally of overall lower quality due to degradation of the starting material. Reflecting the sample size difference, overall replication was higher across the board in the blood data set. Further, the degraded nature of the starting brain RNA-Seq material was reflected in the need for extensive processing of the sequencing reads due to poor library quality. Accordingly, sample outlier removal proved essential in the brain data set, but did not make a meaningful difference in the blood data. While the blood data set was less sensitive to the presence of outliers, this work demonstrates that despite the use of a degraded starting product, biologically meaningful data was still generated from the brain data and that careful data analysis can augment the information garnered from a limited data set. These distinctions between the two independent data sets furthers the point that each RNA-Seq experiment is unique and carries its own limitations, but eQTL replication can be used to guide one’s analysis pipeline.

Finally, it is important to note that our eQTL approach was more helpful in some comparisons than others. While this approach can greatly help to guide one’s analysis, there will be cases where the choice is not so obvious and further steps will need to be taken to assess one’s data processing. For example, when comparing the three data decomposition methods (PCA, SVA, and ISVA) (see Additional file 1: Tables S2 & S3), the answer was unclear, as all methods do a similar job accounting for the unknown covariates. Thus, the choice between the methods was based on additional criteria with PCs being excluded due to confounding within the first PC between sample collection site and disease status and SVs due to their overinflated p-values in differential gene expression analysis (see Additional file 1: Figure S6). Additionally, we note that there are several caveats to the use of data decomposition methods. First, when dealing with small sample sizes, including a large number of covariates can lead to overfitting of the data. Second, data decomposition will minimize the ability to detect global differences in gene expression, which may be correlated with one or more of the eigenvectors (e.g. comparisons across tissues would, by necessity, not incorporate PCs).

In addition to this approach not being applicable for all comparisons, we note that RNA-Seq remains an imperfect measure of gene expression. Technical and analytical limitations remain. Cell type heterogeneity and the need for cDNA generation currently result in unavoidable biases in data generation. While single cell RNA-Seq and direct RNA sequencing methods will address these issues, any improvement that further reduces bias in library construction will lead to more accurate gene estimate values, allowing for further protocol improvement. Additionally, improvements in mapping algorithms, normalization procedures, and gene estimate quantification will also aid in reproducibility.

## Conclusion

In recent years, RNA-Sequencing (RNA-Seq) experiments have moved to the forefront of the transcriptomics field becoming the gold standard approach for the study of genome-wide gene expression. While this period has led to protocols that aim to optimize library preparation and computational methods that aid in improved mapping and accurate gene expression estimation, a method to assess downstream data handling approaches was lacking. Here, we offer a framework that utilizes DNA genotypes and RNA-Seq data along with previously published eQTLs to assess possible sample contamination and assess the biologic validity of each data analysis step to ultimately enable confident downstream analyses.

## Methods

### Sample information

#### Brain

Post-mortem brain samples were acquired through the Autism Tissue Program (http://www.atpportal.org), with samples originating from two different sites: the Harvard Brain Tissue Resource center and the NICHD Brain and Tissue Bank at the University of Maryland. Cortical tissue corresponding to Brodmann Area 19 (BA19) was sequenced in 40 controls and 25 autism-affected cases. Among this set of brains, the average age at time of death is similar between cases and controls (22.2 and 21.3 years, respectively), and there is no significant difference in cause of death between the two groups. One sample had fewer than 20,000 sequenced reads (average across all other samples was 109 M reads) and was excluded. The resultant 64 samples were included for study. This study was approved by the IRB of The Johns Hopkins Hospital and conducted in accordance with institutional guidelines.

#### Blood

Sample data were acquired from the NHGRI GTEx project (phs000424.v3.p1) [9]. Whole blood RNA-Seq and genotyping data were available for 162 samples. This data set comprised of 103 males and 59 females with an average age of 49.7 years.

### Genotyping

#### Brain

Each sample was genotyped at ~900,000 SNPs using the Affymetrix 6.0 array calling genotypes using the Birdsuite software package [24]. High quality genotyping was completed for all samples with an average call rate of 99.63% [range: 97.91 to 99.91].

#### Blood

The GTEx project used the Illumina Omni5 array for direct sample genotyping and subsequently imputed with IMPUTE2 [25] using the 1000 Genome phase 1 release reference panel.

### RNA-sequencing

#### Brain

RNA-Seq libraries were prepared from 50 μg of total RNA from postmortem brain obtaining a fraction of purified polyadenylated (polyA) mRNA after two rounds of hybridization with oligo(dT) dynabeads. Standard quality control measures were employed using “no template controls”, “no ligase controls”, and “no adapter controls” in RNA-Seq library preparation. These samples did not demonstrate detectable product by PCR prior to sequencing. This process was followed by random fragmentation to avoid bias at the 3′ end of the transcript. First-strand cDNA synthesis was performed using random primers (Illumina) and SuperScriptII Reverse-Transcriptase (Invitrogen) followed by second strand cDNA synthesis using RNaseH and DNA Pol I (Illumina). Illumina adaptors were ligated to the purified, end-repaired and 3′ adenylated cDNA and 200 bp size-selection of the final product was performed by gel-excision, following the Illumina-recommended protocol. The 200 bp cDNA template molecules were amplified with the adaptor attached by PCR to create the final library. Each library was sequenced on a single lane of the Illumina’s Hiseq 2000 to produce 100 base pair (bp) single-end reads.

#### Blood

RNA-Seq read count data was obtained from the GTEx project, which used a TruSeq library preparation protocol on poly-A selected mRNA to obtain 72 base paired-end sequencing from the Illumina Hiseq 2000.

### Mapping

#### Brain

The number of total reads per lane varied from 26 M to 202 M, with a mean of 109 M. We used in-house Python scripts to map the sequence reads to the genome (hg19) using Bowtie [26] followed by TopHat [27]. To improve mapping, reads were trimmed to remove stretches of terminal As or Ts (N = 3–12) that occurred as a result of the polyA pulldown step. In addition, we removed contaminating adaptor sequences using a Python script, cutadapt (v0.09). Only uniquely mapped reads with a maximum of three mismatches were used to calculate gene expression values. Aligned reads were sorted, indexed and compressed into the BAM format for easy storage and usage in downstream analysis. The number of total mapped reads per lane varied from 2.7 M to 84.2 M, with a mean of 35 M for the 57 samples used in the final analysis. The RNA-Seq reads were mapped to approximately 44,611 Ensembl genes (average 70% reads mapping per sample). For all analyses (save the case where we analyzed CDS only; see Additional file 1: Table S2), we summarized these reads to all exons of genes based on the coordinates on the hg19/GRCh37 gene annotations provided from Ensembl using the python script HTSeq-count (intersection strict). For the CDS only analysis, HTSeq-count (intersection strict) was again used; however, we excluded reads that mapped to coordinates within the 5′ and 3′ UTRs for summarization. In both cases, regardless of quantification method, we then assessed summarized values on a gene-by-gene basis, removing samples whose gene expression values were more than three SD from the mean expression at each gene. After sample outlier removal, the final gene expression data set was pared down to include the 20,717 genes whose log2 gene expression estimates summed across all 57 samples totaled at least 100.

#### Blood

Mapping was carried out by the GTEx consortium [9]. Our data analysis of these data began with the mapped read count values.

### Normalization

Subsequent to mapping, the gene count data was normalized to minimize biases due to gene-length, GC content, and sequencing depths. CQN normalization procedure was carried out with the recommended default setting [17]. EDASeq normalization was completed using the full-quantile, within-lane GC-content normalization procedure as recommended [16].

### Data decomposition

Data decomposition was performed on the log2 scale for those genes with at least ten gene-level counts across all samples. PCA was performed using the procedure implemented in the R function ‘*prcomp*’. SVA was performed on the matrix of the expression counts, after controlling for case–control status, age, sex and site using the ‘sva’ function implemented in the R package ‘*sva*’. ISVs were generated while protecting for case–control status using ‘isvaFn’ function in the ‘*isva*’ package in R. We applied the unsupervised Bayesian factor analysis method implemented in Probabilistic estimation of expression residuals (PEER) on the count gene expression data [13,22]. PEER yields residual expression factors that can be used in downstream analysis.

### Variant calling

Variant Calling was completed using two different genotyping methods: SAMtools v0.1.12 [28] and the Genome Analysis Toolkit v1.0 (GATK) [29]. SAMtools genotype calls were made for each sample individually using the recommended settings (http://samtools.sourceforge.net/mpileup.shtml); however, we excluded indels from these analyses and filtering was done in-house. Multi-sample GATK calls were made according to the suggested Unified Genotyper generic command line (http://www.broadinstitute.org/gatk/gatkdocs/org_broadinstitute_sting_gatk_walkers_genotyper_UnifiedGenotyper.html). The default settings were used except in the cases of standard minimum Phred-scaled confidence, which was increased to 60 to increase output of confident calls, and downsampling coverage, which was set to 250. We extracted genotypes from each method from the output files and assigned rsIDs (dbSNP build 132) using in-house scripts, keeping genotypes for which there were greater than twenty reads in downstream analyses. Genotypes both concordant across the two variant calling methods and present on the Affymetrix Genome-Wide Array 6.0 were used for downstream analyses.

### Simulated sample mixing experiment

SNPs present on the Affymetrix Genome-Wide Array 6.0 that were called concordantly by both SAMtools and GATK were used in these analyses. The genome function in PLINK (v1.07) [30] was used for pair-wise comparisons to verify that, based on pair-wise IBS distance values (DST), the closest sample match for each RNA sample came from its corresponding DNA sample. Samples with low pair-wise concordance (IBS DST <0.89) were assessed further, computing each sample’s Discordance Ratio (DR). A sample’s DR can be calculated by taking the number of SNPs called homozygous at the DNA level but heterozygous at the RNA level divided by the total number of heterozygous RNA calls. Utilizing this metric, we simulated contamination at the RNA-Seq level by choosing eighteen high-confidence BAM files (DR < 0.1) at random. The Picard (http://picard.sourceforge.net, v1.64) command 'DownsampleSam' was then used to randomly sample a subset or reads from these BAM files. We combined RNA-Seq reads from these 18 samples in controlled ratios [10:90, 20:80, 30:70, 40:60, and 50:50] using samtools’ [28] 'merge' command. After controlled mixing of sequencing reads, we carried out variant calling and comparison back to DNA genotypes on these mixed samples as described above. The DR for each intentionally contaminated sample was calculated and the three samples in question were then compared to our intentionally contaminated subset to determine the level of sample contamination present.

### Assembling lists of previously identified eQTLs

#### Brain

We manually curated a list of brain SNPs and their associated genes from two recent publications [7,8]. These lists were generated from Table S6 [8] and Tables S4 & S6 [7] in the previous publications and included SNPs that had a proxy SNP on the Affymetrix Array 6.0 (r^2^ > 0.90) as determined in SNAP with 1000G CEU as a reference population (http://www.broadinstitute.org/mpg/snap). Additionally, we retained eQTLs whose associated SNPs passed default filtering in PLINK, thus keeping SNPs with <10% missing and SNPs with a minor allele frequency > 0.01. We removed eQTLs whose associated genes were not present in our RNA-Seq data as well as duplicate SNP:gene pairs across the studies (defined as SNP:gene pairs with SNPs w/ r^2^ > 0.8). Combining the lists from the two publications and performing the aforementioned filtering, resulted in a list of 909 eQTLs for study (see Additional file 3).

#### Blood

To test for known eQTLs in blood, we generated a list of 538 *cis*-eQTLs initially identified from a lymphoblastoid cell line [31]. From this data set we started with those *cis*-eQTLs with a q-value < 0.01 in the previously published meta-analysis. Known eQTLs for which the genotyped SNP was present in the imputed GTEx genotype data and the gene was present in the GTEx RNA-Seq expression data were included. This resulted in 538 eQTLs for study (see Additional file 4).

### Covariate inclusion in eQTL analyses

Covariates included in each analysis varied but included a subset or combination of known, unknown, and technical artifacts. The known covariates included were age, sex, and either sample collection site (Harvard or Maryland) in the brain data set or cohort (organ donor, postmortem, or surgical) in the blood data set. We utilized four data decomposition methods – independent surrogate variable analysis (ISVA), surrogate variable analysis (SVA), principal component analysis (PCA) and PEER [13,22] – to account for unknown covariates. We included percent coding bases, percent intronic bases, percent mRNA bases, median 3′ bias, percent UTR bases, and AT dropout as the technical sequencing artifacts in our analyses. [See Picard documentation for further explanation of these artifacts, http://picard.sourceforge.net.]

### Detecting inflation in each data set

To assess inflation of p-values, a genome-wide *cis*-eQTL analysis was carried out for each condition in the R package ‘MatrixEQTL’ (v1.6.1) [32]. eQTLs were detected by looking for *cis*-associations among all directly-genotyped SNPs and genome-wide RNA-Seq gene expression data. *cis*-associations were defined as SNP-gene associations in which the tested SNP was localized within 1 Mb of either the 5′ or the 3′ end of the gene. From the p-value distribution of these analyses, the genome-wide inflation factor in each data set (see Additional file 1: Tables S2 & S3) was determined using the R package ‘GenABEL’ [33].

### Replication of previously identified eQTLs

We utilized the curated list of 909 brain eQTLs and 538 LCL eQTLs to detect eQTLs in our brain and blood data sets, respectively. MatrixEQTL (v1.6.1) [32] was used to test for *cis*-associations between the previously-reported SNP genotypes (or proxy SNPs) and corresponding gene expression estimates from the RNA-Seq data. *cis*-associations were defined as above. In each analysis, p-values were adjusted for inflation [34] using the inflation factor estimated from the genome-wide *cis*-eQTL analysis (see Additional file 1: Table S2 and S3). P-values from this analysis were used to obtain q-values using the R package ‘qvalue’ [20] keeping lambda constant at 0.50. Finally, as used previously [23], in order to assess eQTL replication, the π_1_ statistic was calculated from the inflation-adjusted p-values using the ‘qvalue’ package. π_1_, an estimate of the proportion of replicating eQTLs, is defined as 1-π_0_, where π_0_ is the proportion of true null associations. These three statistics (p-value, q-value, and π_1_) were used to assess the need for and success of each quality control step.

### Differential gene expression analyses

A linear regression framework was utilized to identify differential gene expression between 36 controls and 21 cases with site, age, sex and ISVs as covariates.

### Availability of supporting data

Genotyping and RNA-Sequencing data have been submitted to the NIH’s National Database for Autism Research (NDARCOL0002034). Additional scripts developed for these analyses are available upon request from the authors.

## Abbreviations

RNA-Seq: RNA Sequencing; eQTL: Expression Quantitative Trait Locus; GWAS: Genome-wide Association Study; EDASeq: Exploratory Data Analysis and Normalization for RNA-Seq; CQN: Conditional Quantile Normalization; BA: Brodmann Area; SD: Standard deviation; ISVA: Independent Surrogate Variable Analysis; SVA: Surrogate Variable Analysis; PCA: Principle Component Analysis; IBS: Identity by State; DST: IBS Distance; DR: Discordance Ratio; RIN: RNA Integrity Number; UTR: Untranslated Region; CDS: Coding Sequence; GATK: Genome Analysis Toolkit; GTEx: Genotype-Tissue Expression; LCL: Lymphoblastoid Cell Line.

## Competing interests

The authors declare no financial, personal, or professional conflicts of interest.

## Authors’ contributions

DEA, ABW, and SG conceived the study and designed the experiments. ABW performed the library preparation. SG, SEE, and FNA analyzed the data. JSB, ABW and DEA offered advice during analysis. SEE drafted the manuscript. DEA supervised the project. All authors discussed and commented on the manuscript. All authors read and approved the final manuscript.

## Supplementary Material

Additional file 1 Supplementary figures and tables. Click here for file

Additional file 2 Table of differentially expressed genes. Click here for file

Additional file 3 Table of known brain eQTLs. Click here for file

Additional file 4 Table of known LCL eQTLs. Click here for file
