# Supplementary material for: RNA-Seq optimization with eQTL gold standards
Source: BMC Genomics. 2013 Dec 17;14:892. doi: 10.1186/1471-2164-14-892 (PMC3890578; doi:10.1186/1471-2164-14-892)
Supplement: Additional file 1 — Supplementary figures and tables. [file 1471-2164-14-892-S1.pdf]

RNA-Seq optimization with eQTL gold standards Supplemental Material

Shannon E. Ellis[1], Simone Gupta[1], Foram N. Ashar[1], S. Bader[1,2], Andrew B. West[3] & Dan E. Arking[1]

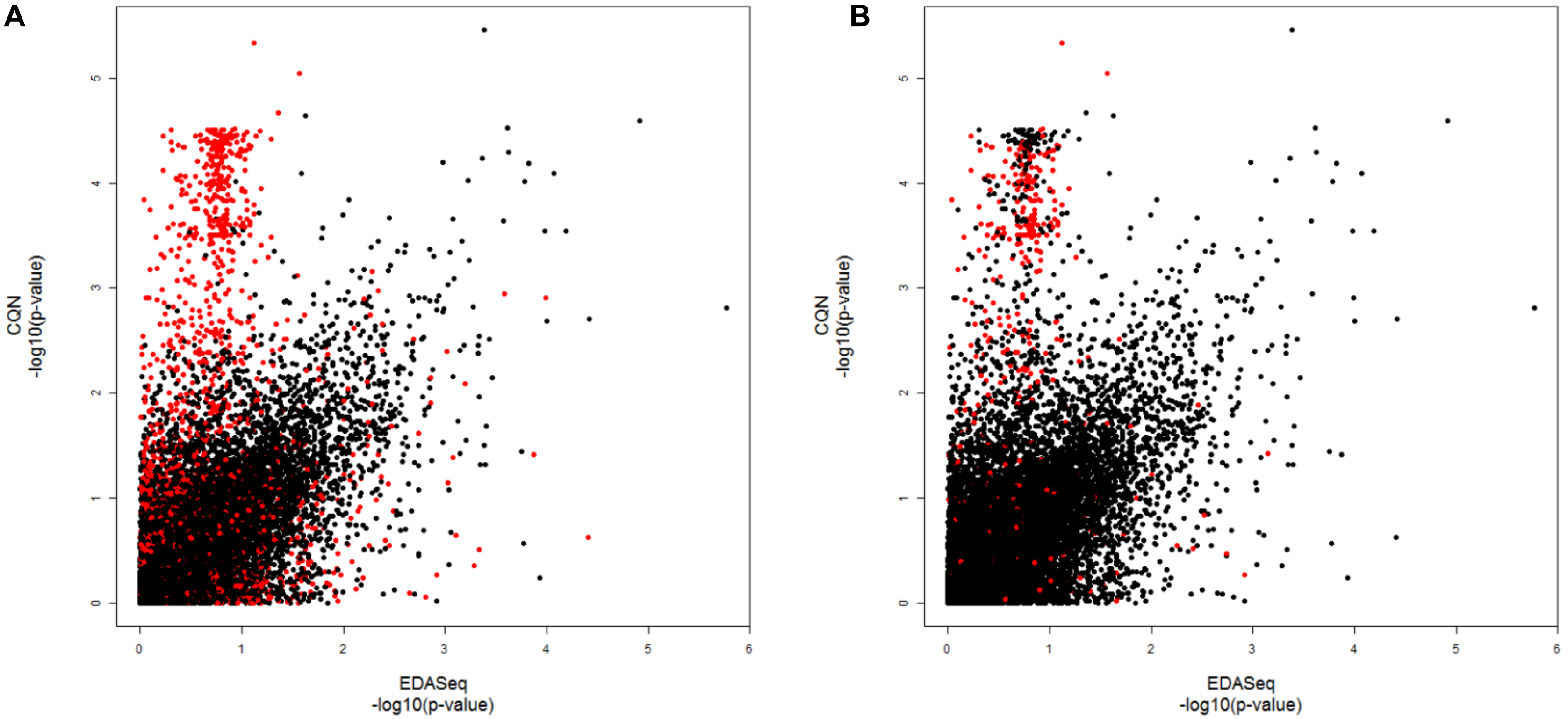

Supplemental Figure 1: **Comparing normalization methods** (**A**) Scatterplot of case-control analysis (–log10) p-values from EDASeq (x-axis) vs CQN (y-axis). Genes with lengths less than 1000 base pairs are highlighted in red. (**B**) Scatterplot of case-control analysis (–log10) p-values from EDASeq (x-axis) vs CQN (y-axis), with genes with GC content < 35% are highlighted in red.

RNA-Seq optimization with eQTL gold standards Supplemental Material

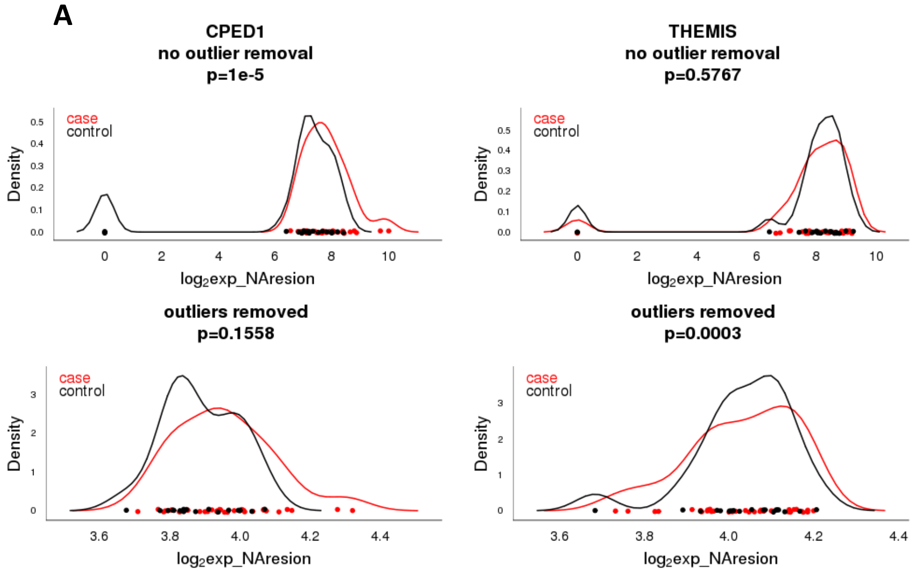

B

		EDASeq		CQN	
		Per gene outliers removed?			
		-	+	-	+
Gene	*CPED1*	$1x10^{-5}$	0.156	0.092	0.094
	THEMIS	0.577	$3x10^{-4}$	0.310	0.065
	RIC8B	0.925	0.197	0.988	0.017
	CHPF	0.022	0.636	0.048	0.992

Supplemental Figure 2: **Sample outliers identified and removed on a gene-by-gene basis improves robustness of differential gene expression analyses (A)** EDASeq gene expression values are plotted. *CPED1,* a false positive, demonstrates a case where the presence of an outlier sample skews the differential gene expression analysis between cases and controls. Prior to outlier removal, the p-value for this gene was $1.04x10^{-5}$; however, by removing the outlier sample, this gene is no longer differentially expressed between cases and controls. On the other hand, *THEMIS* shows the opposite trend in which the comparison demonstrates a more significant difference between cases and controls (p-value from 0.58 to 0.0003) after outliers are removed. **(B)** Differential gene expression p-values before and after per-gene outlier removal in EDASeq and CQN. *CPED1* and *THEMIS* summarize the data presented above in both EDASeq and CQN. In the CQN data, *RIC8B* and *CHPF* are comparable to *THEMIS* and *CPED* in the EDASeq analyses; however, the difference in p-value before and after per-gene outlier removal is less, due to CQN's better handling of outlier samples.

RNA-Seq optimization with eQTL gold standards Supplemental Material

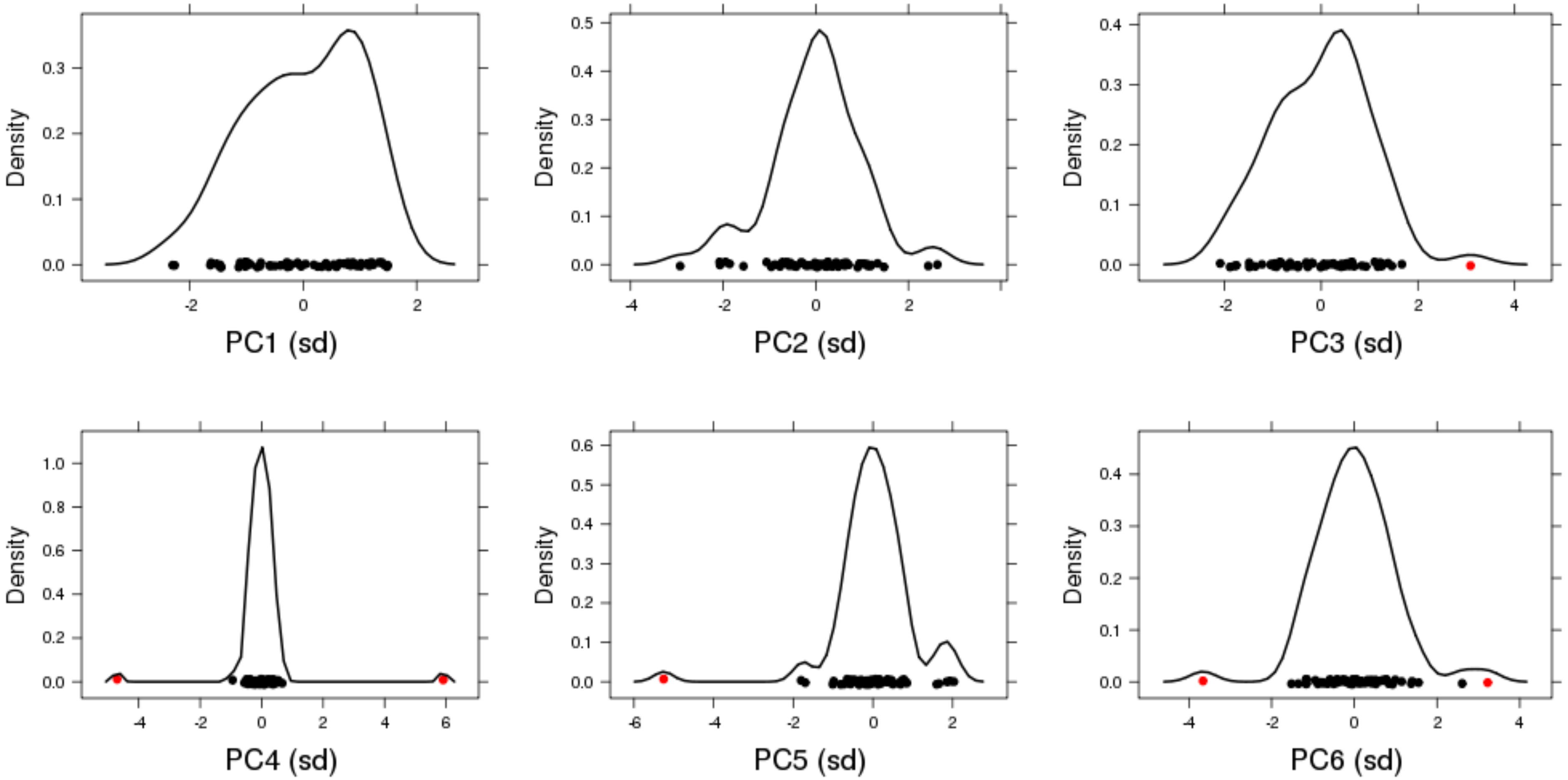

Supplemental Figure 3: **PCA Identifies Sample Outliers**. The first six principle components (PCs) were assessed. Those samples whose gene expression profiles placed them greater than three standard deviations (sd) away from the mean of any of the first six PCs were identified (red) as sample outliers and removed from downstream analyses.

RNA-Seq optimization with eQTL gold standards Supplemental Material

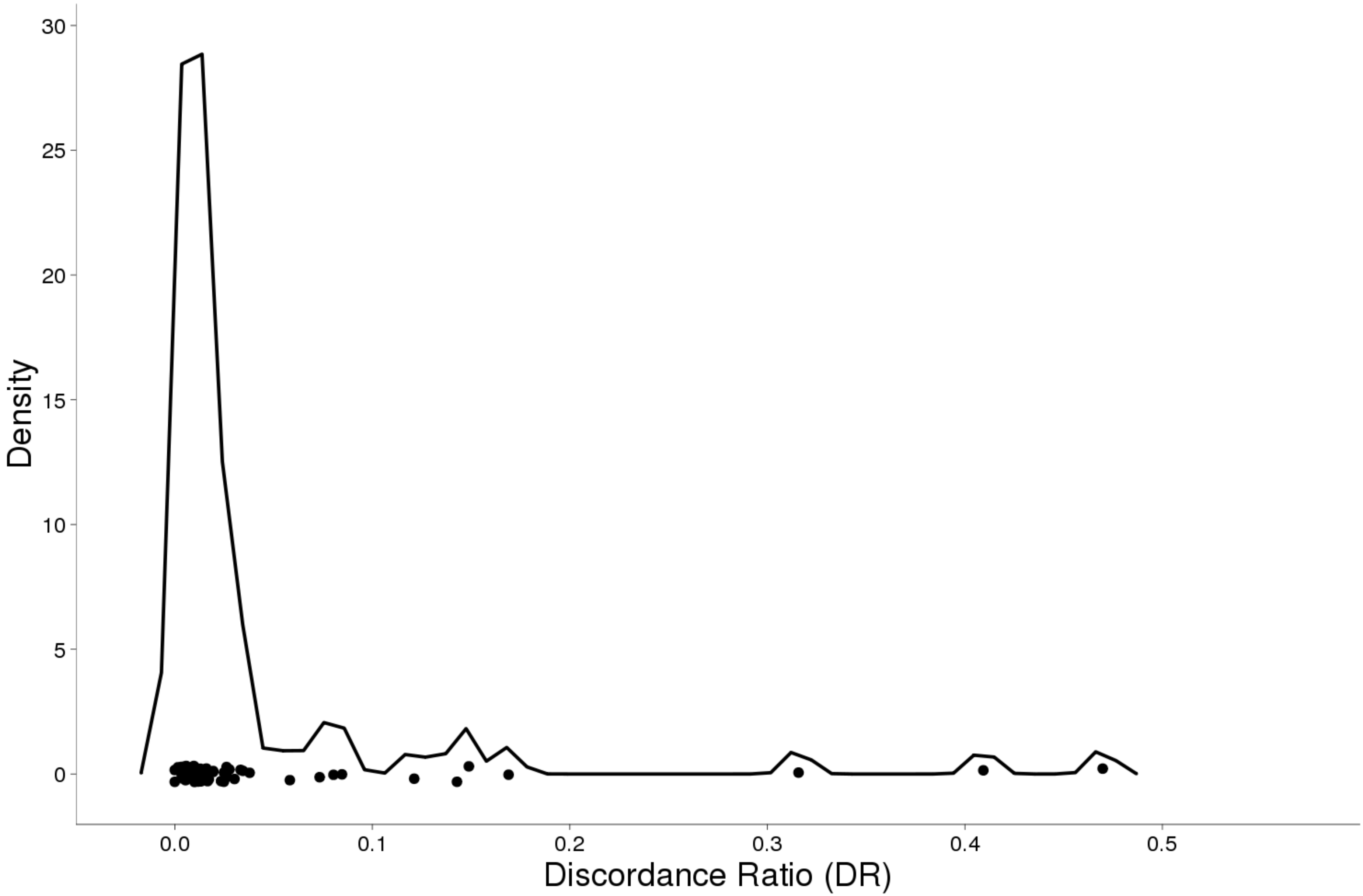

Supplemental Figure 4: Using Discordance Ratio (DR) to assess quality of RNA-Seq data Densityplot of RNA-Seq Samples' Discordance Ratios (DR). A sample's DR can be calculated by taking the number of SNPs called homozygous at the DNA level but heterozygous at the RNA level divided by the total number of heterozygous RNA calls.

RNA-Seq optimization with eQTL gold standards Supplemental Material

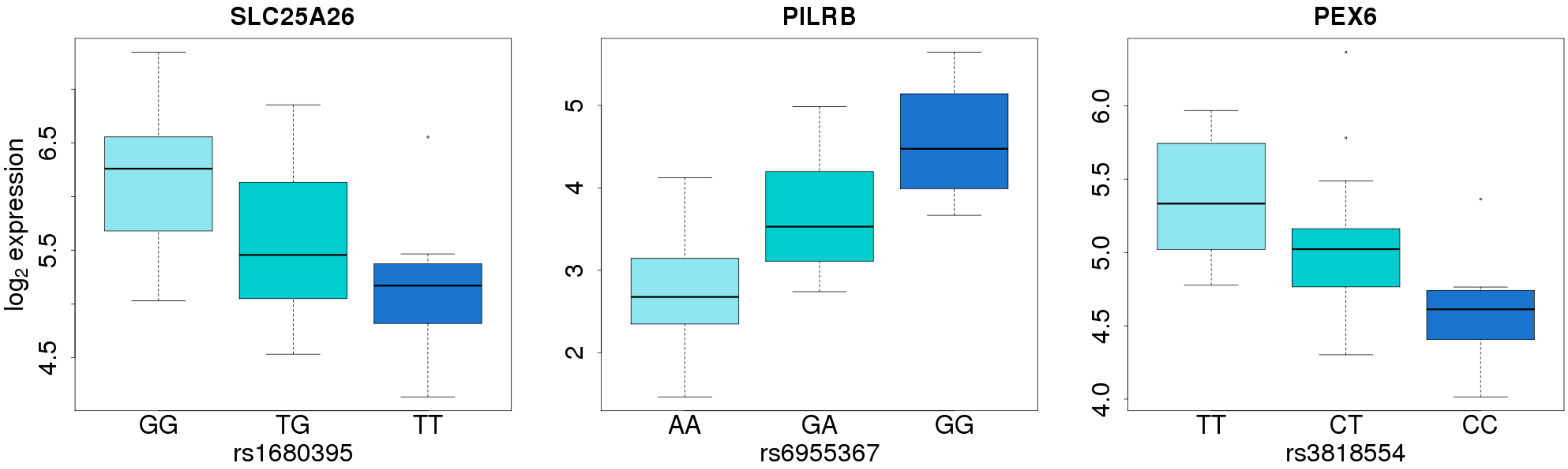

Supplemental Figure 5: Replication of previously-reported eQTLs. A representative set of three eQTLs are shown. Despite a smaller sample size and data from only one brain region, 26.1% of the previously published eQTLs are replicated in our combined dataset at $p<0.05$.

RNA-Seq optimization with eQTL gold standards Supplemental Material

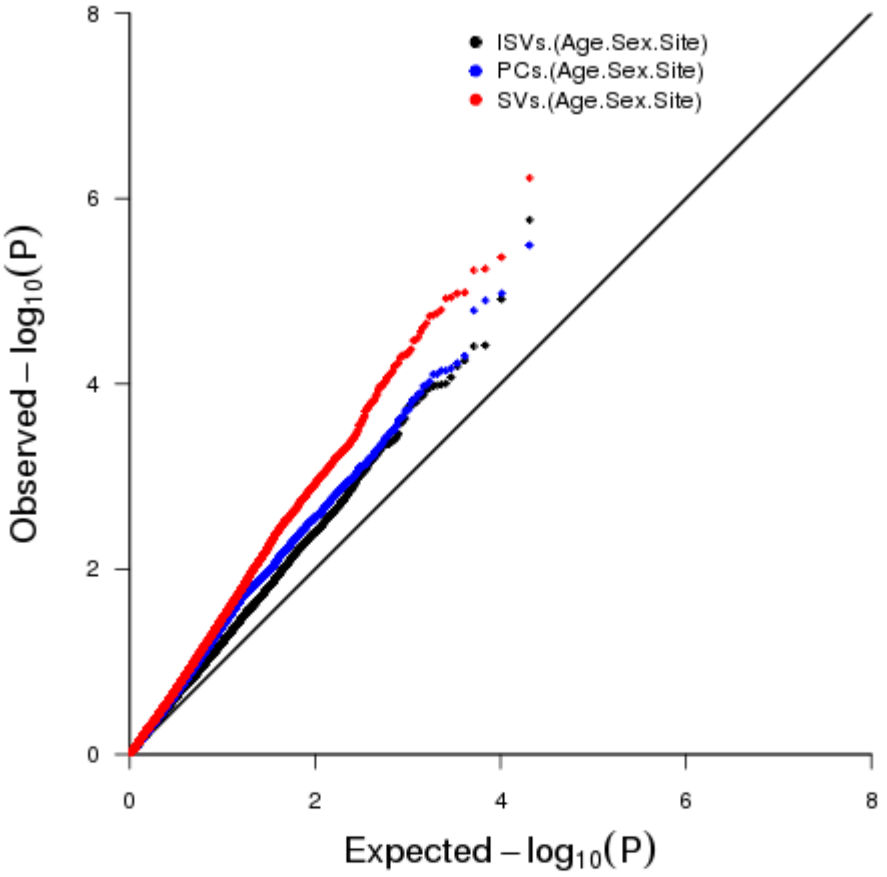

Supplemental Figure 6: **QQ Plots for Data Decomposition Methods**. The deviation from the expected in this QQ-Plot demonstrates inflation in these data, with SVA (red) demonstrating the most significant overinflation among the three methods of data decomposition. This finding supported using ISVs over SVs in our analyses.

RNA-Seq optimization with eQTL gold standards Supplemental Material

Supplemental Table 1: Sample Information

FID	Diagnosis	Sex	Age (y)	Total Mapped Reads	Reason Removed from RNA-Seq Analysis?
AN16641	Autism	M	9	21,584,033	
AN00493	Autism	M	27	4,475,274	
AN00764	Autism	M	20	14,019,291	
AN08792	Autism	M	30	8,226,165	Sample Contamination
AN08873	Autism	M	5	16,702,649	Sample Contamination
AN19511	Autism	M	8	15,225,859	
AN01570	Autism	F	18	29,208,145	
AN09730	Autism	M	22	28,008,384	Sample Contamination
AN17777	Autism	F	49	27,039,497	
AN12457	Autism	F	29	36,768,708	
AN11989	Autism	M	30	34,359,536	
AN13872	Autism	F	5	35,951,442	
AN17678	Autism	M	11	14,071,311	
AN04682	Autism	M	15	3,687,486	
AN03632	Autism	F	49	44,055,322	
AN09714	Autism	M	60	42,351,515	
AN10606	Control	M	56	30,737,028	PC Outlier
AN16665	Control	M	36	19,701,091	
AN01357	Control	M	42	11,514,063	
AN17425	Control	M	16	46,259,333	
AN14368	Control	M	22	2,691,397	
AN15566	Control	F	32	14,500,662	
AN13295	Control	M	56	39,663,314	
UMB797	Autism	M	9	28,573,237	
UMB1349	Autism	M	5	24,697,811	
UMB1638	Autism	F	20	15,048,023	
UMB4231	Autism	M	8	28,320,026	
UMB4721	Autism	M	8	8,185,563	
UMB4999	Autism	M	20	31,898,006	

RNA-Seq optimization with eQTL gold standards Supplemental Material

UMB4671	Autism	F	4	6,400,001	
UMB451	Control	M	4	58,835,797	
UMB497	Control	M	12	33,006,185	
UMB662	Control	F	12	31,463,798	PC Outlier
UMB1185	Control	M	4	84,199,950	
UMB1377	Control	F	5	43,408,008	
UMB1500	Control	M	6	10,489,739	PC Outlier
UMB1674	Control	--	--	43,326,242	
UMB4670	Control	M	4	20,869,197	
UMB4898	Control	M	7	39,936,240	
UMB1323	Control	M	16	60,368,546	
UMB1409	Control	M	18	29,316,044	
UMB1429	Control	M	18	34,503,765	
UMB1322	Control	M	16	38,545,892	
UMB1541	Control	F	20	9,049,911	
UMB1543	Control	M	17	27,552,839	
UMB1584	Control	F	18	84,071,976	
UMB1712	Control	M	20	74,381,568	
UMB1790	Control	M	13	39,010,483	
UMB1796	Control	M	16	40,190,591	
UMB1823	Control	M	15	54,627,736	
UMB1841	Control	M	19	41,505,375	
UMB1843	Control	F	15	77,560,381	
UMB1908	Control	M	13	73,928,312	
UMB4590	Control	M	20	42,752,898	
UMB4591	Control	F	16	42,515,424	
UMB4669	Control	M	16	42,895,894	
UMB4727	Control	M	20	49,847,307	
AN01093	Autism	M	56	20,360,058	PC Outlier
AN06420	Autism	M	39	36,810,746	
		**MIN**	4	2,691,397	
		**MAX**	68	84,199,950	
		**MEAN**	22	33,831,250	

RNA-Seq optimization with eQTL gold standards Supplemental Material

Supplementary Table 2: Summary of eQTL replication analyses carried out in brain samples

Normalization Method	Sample Size (N)	Gene Annotation	Per Gene Outliers Removed?	Covariates	% detected $p<0.05$*	π_1*	% detected $q<0.05$*	Inflation factor (λ)*
EDASeq	64	WG	No	no covariates	10.2%	0.000	1.0%	1.412
EDASeq	60	WG	No	no covariates	10.7%	0.062	1.4%	1.462
EDASeq	57	WG	No	no covariates	17.5%	0.209	3.5%	0.988
EDASeq	57	CDS	No	no covariates	12.1%	0.114	1.7%	1.137
CQN	57	WG	No	no covariates	20.2%	0.217	4.6%	0.968
CQN	57	WG	No	known covariates (age.sex.site)	19.6%	0.241	5.7%	0.966
CQN	57	WG	No	Technical Artifacts	22.6%	0.308	7.8%	0.946
CQN	57	WG	No	PCs	26.1%	0.316	10.8%	0.981
CQN	57	WG	No	ISVs	25.1%	0.327	10.1%	0.965
CQN	57	WG	No	SVs	26.2%	0.290	9.9%	0.960
CQN	57	WG	No	PEER	26.9%	0.321	10.0%	0.955
CQN	57	WG	Yes	PCs	26.1%	0.316	10.1%	0.972
CQN	57	WG	Yes	ISVs	25.7%	0.303	10.3%	0.952
CQN	57	WG	Yes	PEER	25.7%	0.279	11.5%	0.968

*Colors correspond to Figure 2 of the manuscript

RNA-Seq optimization with eQTL gold standards Supplemental Material

Supplementary Table 3: Summary of eQTL replication carried out in GTeX blood samples

<table>
<tr><th>Normalization Method</th><th>Sample Size (N)</th><th>Gene Annotation</th><th>Per Gene Outliers Removed?</th><th>Covariates</th><th>% detected p<0.05*</th><th>π_1*</th><th>% detected q<0.05*</th><th>Inflation factor (λ)*</th></tr>
<tr><td rowspan="2">EDASeq</td><td>162</td><td rowspan="7">WG</td><td rowspan="5">No</td><td rowspan="3">no covariates</td><td>29.6%</td><td>0.374</td><td>17.8%</td><td>1.182</td></tr>
<tr><td rowspan="6">158</td><td>28.3%</td><td>0.387</td><td>16.9%</td><td>1.188</td></tr>
<tr><td rowspan="5">CQN</td><td>31.8%</td><td>0.435</td><td>21.9%</td><td>1.192</td></tr>
<tr><td>known covariates (age.sex.cohort)</td><td>30.3%</td><td>0.416</td><td>19.1%</td><td>1.305</td></tr>
<tr><td rowspan="2">PCs</td><td>41.1%</td><td>0.550</td><td>29.7%</td><td>1.297</td></tr>
<tr><td rowspan="2">Yes</td><td>40.5%</td><td>0.550</td><td>29.9%</td><td>1.268</td></tr>
<tr><td>PEER</td><td>41.1%</td><td>0.550</td><td>32.3%</td><td>1.269</td></tr>
</table>

*Colors correspond to Figure 4 of the manuscript

RNA-Seq optimization with eQTL gold standards Supplemental Material

Supplemental Table 4: Technical Artifacts are correlated with Independent Surrogate Variables. Coefficients greater than 0.45 are bold for emphasis.

Technical Artifact	Correlation Coefficient (r) ISV1	ISV2	ISV3	ISV4	ISV5	ISV6	ISV7	ISV8
**percent coding bases**	0.34	-0.28	0.41	**0.78**	-0.36	**-0.54**	**-0.51**	**0.5**
**percent intronic bases**	-0.03	0.38	**-0.55**	**-0.66**	**0.61**	**0.59**	0.23	**-0.56**
**percent mRNA bases**	0.41	-0.25	**0.45**	**0.48**	**-0.61**	**-0.63**	-0.22	**0.46**
**median 3' bias**	-0.19	0.18	-0.25	**-0.79**	0.09	0.31	**0.57**	-0.32
**percent UTR bases**	0.19	0	0.15	-0.34	**-0.48**	-0.24	0.39	0.02
**AT dropout**	**0.64**	-0.14	0.05	0.19	-0.03	-0.26	**-0.74**	0.18
